# Direct and indirect costs of idiopathic inflammatory myopathies in adults: A systematic review

**DOI:** 10.1371/journal.pone.0307144

**Published:** 2024-07-26

**Authors:** Eden Daniel, Ian C. Smith, Valentina Ly, Pierre R. Bourque, Ari Breiner, Hanns Lochmuller, Nancy Maltez, Kednapa Thavorn, Jodi Warman-Chardon

**Affiliations:** 1 Ottawa Hospital Research Institute, Ottawa, Ontario, Canada; 2 Faculty of Medicine, University of Ottawa, Ottawa, Ontario, Canada; 3 Library, University of Ottawa, Ottawa, Ontario, Canada; 4 Department of Medicine, Neurology, The Ottawa Hospital, Ottawa, Ontario, Canada; 5 Children’s Hospital of Eastern Ontario and Research Institute, Ottawa, Ontario, Canada; 6 Department of Medicine, Rheumatology, The Ottawa Hospital, Ottawa, Ontario, Canada; 7 School of Epidemiology and Public Health, University of Ottawa, Ottawa, Ontario, Canada; Universal Scientific Education and Research Network, UNITED KINGDOM OF GREAT BRITAIN AND NORTHERN IRELAND

## Abstract

Idiopathic inflammatory myopathies (IIMs) are rare disorders characterized by inflammation of skeletal muscle, which can result in fatty replacement of muscle, muscle atrophy, and subsequent weakness. Therapeutic advancements have improved clinical outcomes but impose an economic impact on healthcare systems. We aimed to summarize the direct and indirect costs associated with IIMs in a systematic review (PROSPERO Registration #CRD42023443143). Electronic databases (MEDLINE, Embase, CINAHL, and Scopus) were systematically searched for full-length articles (excluding case reports) reporting costs specific to patients diagnosed with an IIM, published between database inception and April 19, 2023. Direct cost categories included inpatient, outpatient, medication, home/long-term care, and durable medical equipment such as mobility and respiratory aids. Indirect costs included lost productivity. Eligibility criteria were met by 21 of the 3,193 unique titles identified. Costs are expressed in 2023 United States of America dollars, with adjustments for differences in purchasing power applied to currency conversions. As no study reported on all cost categories, annualized cost of IIM per patient was estimated by calculating the mean cost per category, and then adding the means of the different cost categories. By this method, IIM was estimated to cost $52,210 per patient per year. Proportional contributions by category were lost productivity (0.278), outpatient care (0.214), medications (0.171), inpatient care (0.161), home/long-term care (0.122), and durable medical equipment (0.053). Newer findings with intravenous immunoglobulin considered first line therapy for IIM demonstrated markedly higher annual medication costs per patient, upwards of $33,900 compared to an average of $3,908 ± $1,042 in older studies. Future cost-effectiveness studies require updated cost-of-illness studies reflecting the evolving sub-classification and treatment options for IIM, and should consider the impact of IIM on patients and their families.

## Introduction

Idiopathic inflammatory myopathies (IIMs) are a group of rare muscle disorders with an estimated prevalence between 2.4–33.8 cases per 100,000 [1]. IIMs are generally characterized by skeletal muscle inflammation which when associated with muscle damage, can result in muscle fatty replacement, atrophy, and muscle weakness [2]. While muscle weakness is the most recognized manifestation, IIMs are often multisystem disorders with involvement of the lungs, joints, skin, gastrointestinal tract, and heart [3]. IIM places substantial burden on patients, requiring frequent healthcare visits [4], increased need for assistive devices [5], reduced capacity to participate in the labor force [6], increased need for assistive care [7], and reduced quality of life [8]. IIMs also impart significant costs on healthcare systems [4, 9, 10]. For example, there is evidence to support the use of a combination of corticosteroid, immunosuppressive treatment, and immunoglobulin (Ig) delivered intravenously (IVIg) or subcutaneously (SCIg) [2, 11], however, the costs of Ig are substantial [12, 13], and can exceed $250,000 (Canadian) per treatment course [14]. In most cases, IIM does not have monophasic disease course and treatments are required long term [7, 15].

Given the complexity of IIM and the increasing constraints on healthcare systems worldwide, it is important to elucidate the financial implications of IIMs. Further, insights into the economic burden can inform policy development and guide interventions that aim to improve the care for patients with IIMs, particularly with recent advancements in targeted immunotherapies [16, 17]. However, currently, there is no study that comprehensively summarizes the costs associated with IIM. This systematic review was therefore conducted to summarize the published costs of IIM to healthcare systems and society.

## Materials and methods

### Design

This systematic review was reported according to the Preferred Reporting Items for Systematic Review and Meta-Analysis (PRISMA) checklist [18] (S1 Table) and registered on PROSPERO (registration number: CRD42023443143).

### Searched databases and search strategy

A search was carried out on MEDLINE (Ovid), Embase (Ovid), CINAHL (EBSCOhost), and Scopus. Search dates spanned from the date of database inception to April 19, 2023, the date of last search. The main search concepts comprised of terms related to IIMs and economics. The economic concept was informed by the Canadian Agency for Drugs and Technology in Health (CADTH) economic evaluations and models search filters for MEDLINE [19], Embase [20], and CINAHL [21], and adapted for Scopus (S2–S5 Tables). Search results were exported to a web-based reference screening software (Covidence; Melbourne, Australia).

### Eligibility criteria

Studies were included if they met the following criteria: i) the population included adult patients (≥18 years) diagnosed with IIMs; and ii) reported costs associated with IIMs (outlined below). Eligible terms referring to IIM subtypes included dermatomyositis (DM), polymyositis (PM), inclusion body myositis (IBM), necrotizing autoimmune myopathy, myositis-associated with anti-synthetase syndrome, interstitial myositis, and overlap syndromes featuring an IIM with another rheumatological condition [2]. Works were excluded when i) patients with IIM were pooled inseparably with patients with other conditions, ii) the work in question was a case study, case series, conference abstract, or contained no novel data or novel data synthesis from pre-existing data, iii) works were not in English or French, and iv) costs were associated with investigational products (*i*.*e*. drug trials sponsored by a pharmaceutical company).

### Screening procedure

After importing titles and abstracts into Covidence screening software, titles were checked for duplicate entries using the platform’s automated duplicate identification and removal feature. Two reviewers (ED & ICS) independently screened the titles and abstracts within Covidence, and a third reviewer (JWC) resolved any conflicts. Titles and abstracts identified as potentially eligible advanced to the next step which began with identification and import of full texts (if applicable) into Covidence. Imported full texts underwent further eligibility screening. As studies that advanced beyond the title and abstract screening stage required agreement on reason for rejection within Covidence, a reason-for-rejection hierarchy was developed to minimize conflicts. The hierarchy was as follows: conference abstract, letter to editor (no novel data), review article (no novel data synthesis), single case study, case series, article not in English or French, no cost analysis, costs not reported for IIMs, costs not specific to IIMs, cost attributed to an investigational product, could not locate full text. Conflicts were resolved through discussion between ED, ICS, and JWC until a unanimous opinion was achieved. Eligible studies proceeded to the data extraction stage.

### Data collection and management

Data were extracted independently by two reviewers (ED and ICS), using a pre-piloted data extraction form (S1 File). Conflicts were settled by discussion until a unanimous opinion was achieved. Study characteristics extracted included the year of publication, study design, perspective of analysis, data source, and timeline of the source data generation/collection. Participant characteristics extracted included type(s) of IIM studied, age, and sex. Cost data collected included the reporting currency and year, direct costs (subdivided as inpatient, outpatient, emergency department/urgent care, medication(s), and home/long term care/other), indirect costs (subdivided as absenteeism, presenteeism, lost productivity, and non-work impairments), and the country in which the reported costs were incurred. Direct healthcare costs without an assigned monetary value such as length of hospital stay, number of hospitalizations, and number of visits to clinics were also collected.

### Synthesis of results

To enhance the compatibility of cost data, all costs were inflated and converted to 2023 USA dollars (USD) using the Campbell and Cochrane Economics Methods Group Evidence for Policy and Practice Information Coordinating Centre (CCEMG-EPPI-Centre) Cost Converter web tool [22]. This web tool first adjusts costs from the original price-year to the target price-year, and then converts from the original currency to target currency using conversion rates which include adjustments for differences in purchasing power between nations. When reported costs were expressed in a currency other than that in which costs were incurred but did not adjust for difference in purchasing power, costs were converted back to the original currency using either the conversion rate stated in the original manuscript, or the mean annual conversion rate published by the International Monetary Fund [23], and then entered into the CCEMG-EPPI-Centre Cost Converter web tool.

Studies were described in terms of country/countries of study, and types of cost reported. With exception of total reported costs, subcategorizations of IIM patients (e.g., by sex, time with condition, modality of treatment) were collapsed using *n*-weighted averaging. The different IIM disease subtypes were not collapsed in this systematic review and were rarely encountered in the studies that met eligibility criteria. When possible, we also categorized costs as direct and indirect costs. Direct costs refer to expenses directly associated with medical care, such as hospital stays, medications, medical procedures, and physician visits. Indirect costs encompass non-medical expenses related to a disease, including lost productivity, time off work, and other out-of-pocket expenses incurred by patients and caregivers. An aggregated estimate of the annualized cost per person with IIM was determined by calculating the mean of each cost category and then summing of the mean of each cost category.

### Quality assessment and risk of bias checklists

The Consolidated Health Economic Evaluation Reporting Standards (CHEERS) checklist [24] was used to assess the reporting quality of the included studies. The risk of bias assessment was conducted using the Joanna Briggs Institute Critical Appraisal Checklist for Economic Evaluations [25]. Assessments were conducted independently by ED and ICS with conflicts settled by discussion.

## Results

**Fig 1:**
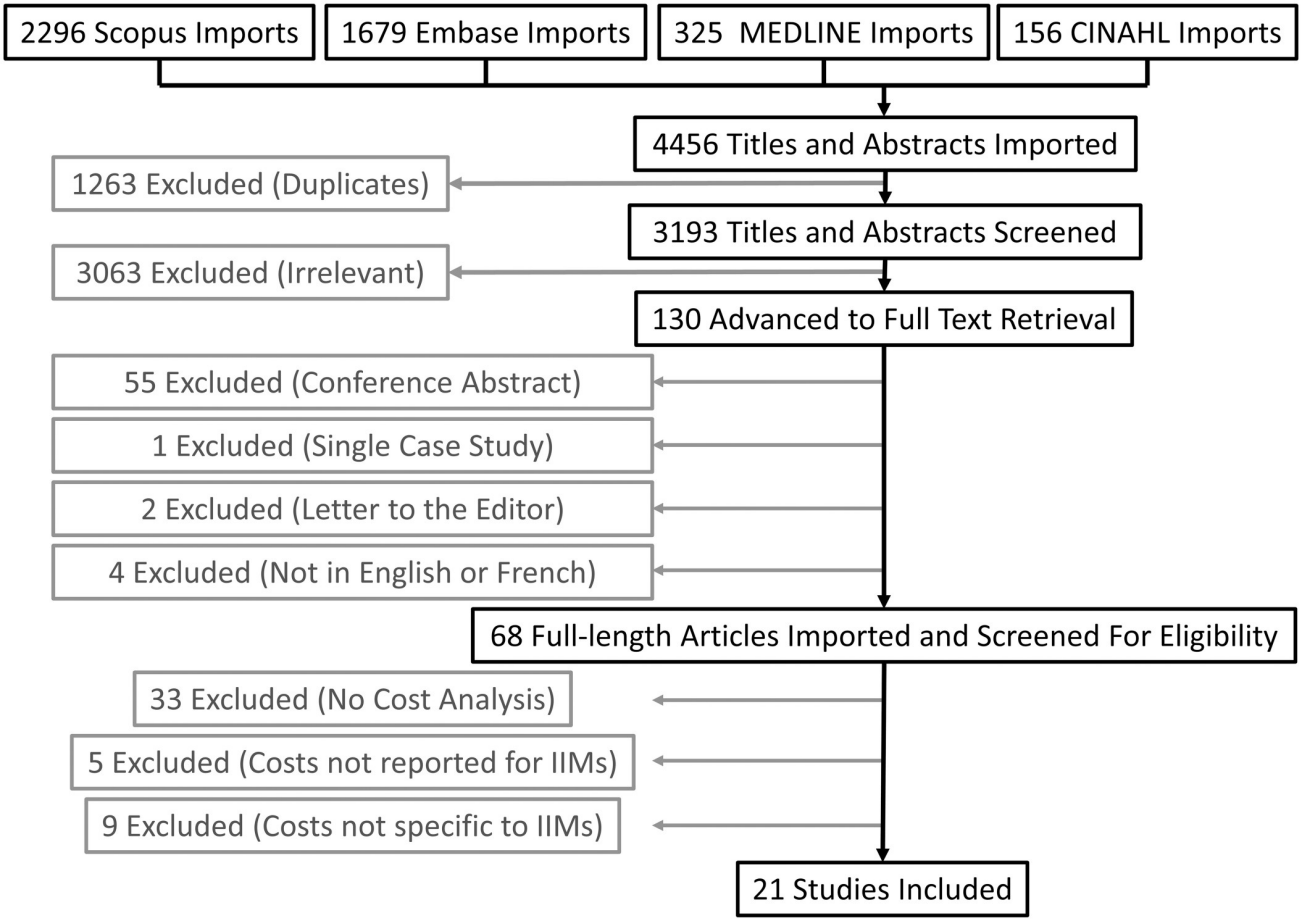
PRISMA flow chart.

The search identified 4456 titles, including 1263 duplicates. Out of 3193 unique titles identified, 130 titles advanced to the full text retrieval stage, 68 titles advanced to the full-length screening stage, and 21 studies met eligibility criteria and advanced to data extraction and full inclusion. A PRISMA flow chart is shown in Fig 1.

### Characteristics of included studies

Fifteen of the 21 included studies were conducted in North America (14 in USA (_((((xxx))))_)[4, 26–38] and 1 in Canada [39]), 3 in Asia (2 in Thailand [40, 41] and 1 in Japan [42]), 1 in Sweden [9]), 1 in Australia [43], and 1 was a Cochrane review [44] (Table 1). All represented countries are categorized as having very high human development by the United Nations Development Programme (range in human development index = 0.803–0.952, contrasted with the global average of 0.739; 2022 values) [45]. Apart from one article published in 1995 [35], all included studies were published after 2010, with a median year of publication of 2017. Source data were concentrated to the decade spanning from 2005 to 2014 (S1 Fig). Only two studies provided explicit statements regarding the perspectives of the provided cost of illness estimates, one taking the societal perspective [40], and one taking the patient perspective [28]. Eighteen out of 21 studies used databases containing administrative records as primary data source (_((((xxx))))_)[4, 9, 26, 27, 29–42]. All but two studies were retrospective in design. The exceptions were a cost-effectiveness study for MRI-guided biopsy to aid diagnosis [35], and a cost-utility analysis of IVIg in Thailand [40]. The risk of bias within studies was deemed negligible to the scope of the present review (S6 Table). However, no study reported on all cost categories, so the generalizability of studies to the setting of interest for this review was always considered limited. CHEERS reporting quality checklists are summarized in S7 Table.

**Table 1:** Characteristics of included studies.

Study	Original Currency	Country and Period of Study	IIMSubtype(s)	Study Population (n, % Female, mean age)	Study Design	Data Source
Bamrungsawad et al. 2015 [40]	USD 2012^†^	Thailand 2012*	DM	Hypothetical cohort (*i*.*e*., simulated data) 40+ years	Cost-utility analysis using a Markov model to estimate costs	National Health Security Office, Drugs and Medical Supplies Info Centre, Hospital Database, 2 Neurologists and a Health Economist
Bernatsky et al. 2011 [39]	CAD 2008	Canada 1989–2003	DM/PM	n = 1102 68.9% Female 57.4±18.4 years	Retrospective database analysis of direct costs	Physician billing and hospitalization databases of Quebec
Bradford Rice et al. 2016 [26]	USD 2013	USA 1998–2014	DM/PM	n = 2617 64.6% Female 49.5±10.5 years	Retrospective database analysis of direct and indirect costs	Administrative claims from OptumHealth Reporting and Insights
Capkun et al. 2017 [27]	USD 2016*	USA 2009–2013	IBM	n = 333 34% Female 69±9.6 years	Retrospective database analysis of direct costs	Truven Health MarketScan Commercial Claims and Encounters and Medicare Supplemental Research database
Christopher-Stine et al. 2020 [28]	N/A	USA 2017	DM/PM	n = 524 78.1% Female 55.4±12.9 years	Cross-sectional survey of indirect costs and unplanned medical encounters	Patient surveys via The Myositis Association and John Hopkins Myositis Center
Foocharoen et al. 2013 [41]	USD 2010*^‡^	Thailand 2010	DM/PM	n = 269 61.7% Female 50.4±13.7 years	Cross-sectional study of hospitalization rates and direct costs	National pooled database of hospitalized patients
Foreman et al. 2017 [43]	AUD 2016*	Australia 2000–2014	DM/PM/IBM	n = 57 70% Female 58 (24–87) years	Retrospective case-note review of IVIg costs	Case-notes from physician assessments in South Australia
Furst et al. 2012 [29]	USD 2009	USA 2003–2008	DM/PM/Interstitial Myositis	n = 4,487 66.0% Female 55% were 45–64 years	Retrospective database analysis to assess resource utilization longitudinally in insured patients	Managed Care Organization database affiliated with OptumInsight
Keshishian et al. 2018 [4]	USD 2014	USA 2009–2013	IBM	n = 361 47.6% Female 75.8±6.3 years	Retrospective database analysis of direct costs	National Medicare database
Knight et al. 2017 [30]	USD 2015*	USA 2009–2014	DM/PM	n = 1,967 68.9% Female 48.5 years	Retrospective observational database study comparing non-medication costs	Commercial health insurance administrative claims databases
Kundrick et al. 2019 [31]	USD 2016	USA 2005–2014	DM	n = 6.3 x10^6^ scans** 72.6% Female 0–64 years	Retrospective comparison of costs of different modalities for pulmonary and malignancy screening.	MarketScan Commercial Claims and Encounters Database
Kwa et al. 2017 [32]	USD 2014	USA 2002–2012	DM	n = 11,092 hospitalizations 71.6% Female 58.1 years	Retrospective database analysis examining inpatient cost of care	National Inpatient Sample from Agency for Healthcare Research and Quality
Leclair et al. 2021 [9]	EUR 2019	Sweden 2010–2016	DM/PM/IBM	n = 673 61% Female 60±16 years	Population-based longitudinal cohort study estimating annual direct and indirect costs 5 years pre- and post-diagnosis	Swedish National Patient Register
Miyazaki et al. 2021 [42]	JPY 2019*	Japan 2009–2019	DM/PM	n = 836 60.4% Female 46.9±15 years	Retrospective longitudinal examination of healthcare costs in the three years following diagnosis	Health insurance claims data retrieved from Japan Medical Data Center database
Ren et al. 2019 [33]	USD 2009	USA 2002–2012	DM	n = 58,587 admissions 73.7% Female 57.4 years	Retrospective examination of inpatient costs with/without serious infection	National Inpatient Sample from Agency for Healthcare Research and Quality
Rosas et al. 2021 [34]	USD 2020*	USA 2005–2014	DM/PM	n = 545 74.9% Female Median ~70 years	Retrospective examination of cost of total hip arthroplasty	National database of surgeries and outcomes
Rose et al. 2015 [44]	GBP 2015*	International 1993–2014	IBM	Not Applicable	Cochrane review assessing the effectiveness of treatments	Cochrane Neuromuscular Disease Group Specialized Register, the Cochrane Central Register for Controlled Trials, MEDLINE, and EMBASE
Schweitzer & Fort 1995 [35]	USD 1994*	USA 1993–1994*	PM	n = 25 28–55 years	Single centre prospective study assessing cost effectiveness of MRI-guided muscle biopsy	Hospital accounting system
Tripathi & Fernandez 2021 [36]	USD 2015	USA 2009–2015	DM	n = 39,253 hospitalizations 73% Female 63% were 40–79 years	Retrospective analysis of inpatient costs with and without malignancies	National Inpatient Sample from Agency for Healthcare Research and Quality
Ungprasert et al. 2020 [37]	USD 2014	USA 2005–2014	DM/PM	n = 160,528 admissions 68.7% Female 58.0 years	Retrospective database analysis examining inpatient costs and resource utilization	National Inpatient Sample from Agency for Healthcare Research and Quality
Zhang et al. 2019 [38]	USD 2014	USA 2010–2014	DM	n = 2016 same-cause readmissions	Retrospective examination of hospitalization and readmission rates and associated costs	Nationwide Readmissions Database

* Year not explicitly reported; year is assumed.

^†^Costs converted back to Thai baht (31.08 per $1 USD) [40].

^‡^ Costs converted back to Thai baht (31.69 per $1 USD) [23].

DM: Dermatomyositis, IBM: Inclusion Body Myositis, IIM: Idiopathic Inflammatory Myopathies, IVIg: Intravenous Immunoglobulin, MRI: Magnetic Resonance Imaging, PM: Polymyositis

### Patient characteristics

Consistent with greater prevalence of IIM in females [46], most studies (_((((xxx))))_)[9, 26, 28–34, 36, 37, 39, 41–43] reported more females than males in their study population (Table 1). The two studies reporting more males than females in their study population examined IBM [4, 27], which is more prevalent in males than in females [46]. Four studies [35, 38, 40, 44] did not report sex ratios. Regarding IIM subtypes, six studies reported on DM alone [31–33, 36, 37, 40], one reported only PM alone [35], eight reported on both DM and PM [26, 28, 30, 34, 37, 39, 41, 42], three reported on IBM alone [4, 27, 44], two reported on DM, PM, and IBM [9, 43], and one reported on DM, PM, and interstitial myositis [29] (Table 1). Mean reported ages ranged from 46.9±15 years for a study of DM/PM patients [42] to 75.8±6.3 years for a study of IBM patients [4].

### Direct costs of IIM

#### Inpatient costs

Inpatient costs were reported by 15 studies (Table 2). Inpatient costs per patient per annum were provided by eight studies, with reported annual costs ranging from $1,158 to $21,928. Annual costs per person with IIM were higher on average for studies from the USA compared to studies from outside the USA ($13,041±$9,065 vs $3,811±$2,088). Seven studies [32, 33, 35–38, 41] provided inpatient costs per hospitalization for IIM (Table 2) which could not be converted to an annual costs per person with IIM. The lowest cost per hospitalization was reported in Thailand, at $5,088 per hospitalization with mean length of stay of 11 days (Table 3) [41]. The remaining six studies [32, 33, 35–38] reporting cost per hospitalization were based in the USA and costs ranged from $11,852 (4 days stay, patients with DM) to $65,615 (7 days stay, patients with DM or PM), and had median cost per hospitalization of $28,843. Cost of hospitalization per day could be determined for five USA-based studies [32, 33, 35–37], four of which used National Inpatient Sample (NIS) as a data source [32, 33, 35–37]. Three studies reported inpatient costs between $2,236 per day and $3,090 per day [32, 33, 35], one study reported inpatient costs of $9,583 per day [36], and one study indicated that cost per day varied according to perspective, with the hospital incurring costs of $2,827 per day, but charging $9,374 [37].

**Table 2:** Categorized direct monetary costs of illness in idiopathic inflammatory myopathies.

Study (Country of Study)	IIM Subtype	Inpatient	Outpatient	Emergency/Urgent Care	Inpatient, outpatient, emergency combined	Medication/Pharmacy	Home/Long Term Care/Other
**Studies reporting annual costs per patient with IIM**							
Leclair et al. 2021 (Sweden) [9]	DM/PM/IBM	$5,685	$3,564		$9,249	$489 (Immunosuppressants) $880 (IVIg) $1,280 (Other)	
Furst et al. 2012 (USA) [29]	DM/PM/Interstitial myositis	$4,828	$9,796	$401	$15,026	$3,735 (Undifferentiated)	$1,589 (Unspecified)
Bernatsky et al. 2011 (Canada) [39]	DM/PM	$3,143	$1,109		$4,252		
Bradford Rice et al. 2016 (USA) [26]	DM/PM				$14,558	$3,257 (Undifferentiated)	
Knight et al. 2017 (USA) [30]	DM/PM	$19,770	$26,520		$59,811		
Miyazaki et al. 2021 (Japan) [42]	DM/PM	$5,256	$7,940		$18,131	$33,909 (Undifferentiated)	
Bamrungsawad et al. 2015 (Thailand) [40]	DM	$1,158	$3,912		$5,070		
Capkun et al. 2017 (USA) [27]	IBM	$5,638	$22,265	$881	$34,941	$5,206 (Undifferentiated)	
Keshishian et al. 2018 (USA) [4]	IBM	$21,928	$12,166	$713	$34,807	$4,695 (Undifferentiated)	$7,233 (Skilled Nursing Facilities) $3,382 (Home Health Agency) $567 (Hospice Care) $2,778 (Durable Medical Equipment)
**Studies reporting annual cost per patient with IIM receiving a specific treatment or type of care**							
Foreman et al. 2017 (Australia) [43]	DM/PM/IBM					$7,546 (Truncated IVIg) $25,798 (Prolonged IVIg)	
Bamrungsawad et al. 2015 (Thailand) [40]	DM					$42,112 (IVIg) $128 (Prednisolone) $651 (Immunosuppressants)	$8,506 (Nursing Home) $588 (Home care–Disabled) $133 (Home care—Pre-Disabled)
Rose et al. 2015 (International) [44]	IBM					$50,511 (IVIg)	
**Studies reporting cost per episode of care**							
Foocharoen et al. 2013 (Thailand) [41]	DM/PM	$5,088					
Ungprasert et al. 2020 (USA) [37]	DM/PM	$19,784 ^a^ $65,615 ^b^					
Schweitzer & Fort 1995 (USA) [35]	PM	$28,843					
Kwa et al. 2017 (USA) [32]	DM	$11,852					
Ren et al. 2019 (USA) [33]	DM	$30,075 ^c^ $16,069 ^d^					
Tripathi & Fernandez 2021 (USA) [36]	DM	$55,581					
Zhang et al. 2019 (USA) [38]	DM	$17,098					
Bamrungsawad et al. 2015 (Thailand) [40]	DM						$13 (Transportation) $4 (Meal) $9 (Companion Income loss)
**Studies reporting cost for a specific procedure**							
Rosas et al. 2021 (USA) [34]	DM/PM		$16,519 ^e^				
Kundrick et al. 2019 (USA) [31]	DM		$1,940 ^f^				

Costs have been converted to 2023 USD with adjustments for purchasing power parity.

^a^ Costs of Hospital;

^b^ Charges by Hospital;

^c^ With serious infection;

^d^ Without serious infection;

^e^ Cost of malignancy and pulmonary screening panels;

^f^ 90-day costs following total hip arthroplasty

IIM: Idiopathic Inflammatory Myopathies, IVIg: Intravenous Immunoglobulin

**Table 3:** Healthcare resource utilization in idiopathic inflammatory myopathies.

Study (Country of Study)	IIM Subtype(s)	Mean Length of Hospitalization (days)	Mean Annual Number of Hospitalizations	Mean Annual Number of Outpatient Visits	Mean Annual Number of Emergency or Urgent Care Visits
Keshishian et al. 2018 (USA) [4]	IBM	7.9*	0.9	11.2	
Furst et al. 2012 (USA) [29]	DM/PM/Interstitial Myositis	2.0†	0.2	27.1	3.0
Leclair et al. 2021 (Sweden) [9]	DM/PM/IBM	5.8†		7.0	
Knight et al. 2017 (USA) [30]	DM/PM	4.1†	1.9	40.9	0.6
Bradford Rice et al. 2016 (USA) [26]	DM/PM	2.2†	2.6	17.3	0.7
Miyazaki et al. 2021 (Japan) [42]	DM/PM	2.7†	0.7	5.6	
Christopher-Stine et al. 2020 (USA) [28]	DM/PM		0.3		0.9
Foocharoen et al. 2013 (Thailand) [41]	DM/PM	11.0†			
Ungprasert et al. 2020 (USA) [37]	DM/PM	7.0†			
Schweitzer & Fort 1995 (USA) [35]	PM	12.9†			
Kwa et al. 2017 (USA) [32]	DM	4.8†			
Tripathi & Fernandez 2021 (USA) [36]	DM	5.8†			
Ren et al. 2019 (USA) [33]	DM	10.2† (With serious infection) 5.2† (Without serious infection)			
Bamrungsawad et al. 2015 (Thailand) [40]	DM		0.3	16.6	

*Per annum

^†^ Per hospitalization

DM: Dermatomyositis, IBM: Inclusion Body Myositis, IIM: Idiopathic Inflammatory Myopathies, PM: Polymyositis

#### Outpatient costs

Outpatient costs per patient per annum were reported by eight studies (Table 2). Outpatient costs ranged widely from $1,109 to $26,520. Mean outpatient costs were lower in studies conducted outside of the USA ($4,131±$2,829 vs $17,687±$10,315; mean ± standard deviation (SD)). Two studies focused on specific outpatient tests and procedures which were deemed to be too niche to be generalized to the context of this review [31, 34], but are listed at the bottom of Table 2.

#### Emergency department/urgent care costs

Emergency department and urgent care costs were reported by three studies [4, 27, 29], all conducted in the USA. The range of mean annualized cost per patient was $401-$881 (Table 2).

#### Combined inpatient, outpatient, and emergency/urgent care costs

The combined inpatient, outpatient, and emergency department costs were either provided by, or calculated for nine studies (Table 2), and ranged from $4,252 to $59,811. Mean costs were higher in studies conducted in the USA than studies conducted outside the USA ($31,829±$18,589 vs $9,176±$6,359).

#### Medication costs

Medication costs were reported in nine studies (Table 2), with six studies reporting the annualized costs of medication per patient [4, 9, 26, 29, 42] and three reporting the cost per patient receiving a specific treatment [40, 43, 44]. Four studies reported costs associated with specific medications [9, 40, 43, 44], and five studies provided a pooled cost of all medications [4, 26, 27, 29, 42]. The highest reported annual drug costs per patient were in Japan at $33,909 where IVIg was considered first-line therapy [42], and the lowest costs were reported in Sweden, at $2,649 [9]. Biologic immunosuppressants were the most expensive drug category specified within individual studies, with the cost to treat one patient ranging from $7,546 [43] to $50,511 [44].

#### Home/long-term care and other costs

Three studies [4, 29, 40] listed costs related to home healthcare, nursing homes, durable medical equipment, direct costs and opportunity costs of patients and companions associated with attending clinical visits, or costs not otherwise specified (Table 2). Annual costs associated with nursing facilities exceeded costs of home-based care when expressed specific to need ($8,506 vs $588 [40]), and when averaged across the population with IIM ($7,233 vs $3,382 [4]). Costs of durable medical equipment were mentioned specifically in only one study, costing $2,778 per person with IIM [4].

#### Healthcare resource utilization

Healthcare resource use by patients with IIM was reported in 14 studies (Table 3). Eleven studies reported the mean length of stay per hospitalization. Across these 11 studies, mean length of stay per hospitalization was 6.1± 3.5 days, with a range of 2.0–12.9 days. The mean number of hospitalizations per patient per year ranged from 0.2 to 2.6, with a mean ± SD of 1.0 ± 0.9 (seven studies reporting). The mean number of outpatient visits per patient per year ranged from 5.6 to 40.9, with mean ± SD of 18.0 ± 12.5 (seven studies reporting). The mean number of emergency or urgent care visits per patient per year ranged from 0.6 to 3.0, with mean of 1.3 and SD of 1.1 (four studies reporting).

### Indirect costs of IIM

Three studies reported indirect costs of IIMs [9, 26, 28] (Table 4). Methodological differences prevent direct comparisons between studies. Leclair et al. [9] reported data from the Swedish National Patient Register, which excludes sick leave of less than two weeks in duration that are covered by employers. In contrast, the data included in the study by Bradford Rice et al. [26] were obtained from self-insured large employers in the USA, predominantly comprised of data from short-term absences. As the indirect cost data provided by Leclair [9] and Bradford Rice et al. [26] seemingly reflect costs of long-term and short-term leaves, respectively, their data were treated as complementary rather than distinct in the calculation of the aggregate cost of IIM (outlined below). Christopher-Stine et al. [28] reported a 9% loss of work time, 22% decline in productivity while working, 28% decline in work productivity, and a 40% impairment of non-work activity due to IIM. Although no dollar values were available for the work-related productivity losses [26, 28], these costs were calculated for this review based on 260 working days per year and median weekly earnings of $1,118 ($58,335 per year) [47]. There was no established cost analysis on the impairment of non-work activity.

**Table 4:** Indirect costs of IIM reported per annum.

Study (Country of Study)	IIM Subtype(s)	Disability	Absenteeism	Other
Bradford Rice et al.2016 (USA) [26]	DM/PM	6.8 Days $1,526†	10.7 days $2,401†	
Christopher-Stine et al. 2020 (USA) [28]	DM/PM		9%* $5,250†	Presenteeism = 22%* ($12,834†) Work Productivity Loss = 28%* ($16,334†) Non-work Activity Impairment = 40%
Leclair et al. 2021 (Sweden) [9]	DM/PM/IBM	29.0 days $4,056	47.8 days $4,791	

Costs have been converted to 2023 USD with adjustments for purchasing power parity.

*Cost provided as a percentage of work time

^†^Calculated within this review based on 260 working days per year and median weekly earnings of $1,118 ($58,335 per year) [47].

DM: Dermatomyositis, IBM: Inclusion Body Myositis, IIM: Idiopathic Inflammatory Myopathies, PM: Polymyositis

### Total reported costs of IIM

Total costs of care for IIM, including medications, were reported by six studies [4, 9, 26, 27, 29, 42] (Table 5). Mean costs varied considerably between studies, ranging from $11,100 for patients with interstitial myositis in the USA [29] to $89,600±$173,100 for patients with DM or PM in Japan [42]. Costs of care also varied considerably for patients within studies, with the standard deviation of costs exceeding the mean cost in most cohorts [9, 26, 42] (Table 5). The lone exception [27] reported cost data with statistical adjustments which prevent the reported standard deviation from being interpreted at face value. Costs of care were higher for patients with a recent diagnosis of IIM than for patients who had been diagnosed years prior [9, 29, 39, 42]. Cost increases were identified to occur before diagnosis was received [29]. Only one study provided costs of different IIM subtypes, reporting that total costs of care were higher in DM than in PM, and higher in PM than in interstitial myositis [29] (Table 5). Leclair et al. [9] reported that the costs associated with IIM-related sick leave and disability exceeded those of the inpatient, outpatient, and medication cost categories for their full cohort of patients. However, since productivity losses were measured as deviations from the expected participation in the labour force, productivity losses were concentrated to the subset of patients below 65 years of age (i.e., those below the typical age of retirement) [9]. Medical costs were higher in those 65 years of age and over versus those below 65 years of age [9].

**Table 5:** Reported total annualized costs of idiopathic inflammatory myopathies.

Study (Country of Study)	IIM Subtype(s)	Total Costs Per Patient
Bradford Rice et al. 2016 (USA) [26]	DM/PM	$17,800±$47,000
Miyazaki et al. 2021 (Japan) [42]	DM/PM (Overall)	$42,800±$82,900
	1 Year Post-Diagnosis	$89,600±$173,100
	2 Years Post-Diagnosis	$14,400±$49,200
	3 Years Post-Diagnosis	$10,400±$33,200
Furst et al. 2012 (USA) [29]	Newly Diagnosed:	
	DM/PM/Interstitial Myositis	$20,900
	DM	$33,600
	PM	$24,100
	Interstitial Myositis	$12,000
	Existing Diagnosis:	
	DM/PM/ Interstitial Myositis	$20,000
	DM	$27,300
	PM	$22,400
	Interstitial Myositis	$11,100
Leclair et al. 2021* (USA) [9]	DM/PM/IBM	$20,800
	5 Years Pre-diagnosis	$5,900±$14,400
	4 Years Pre-diagnosis	$6,600±$14,400
	3 Years Pre-diagnosis	$6,500±$15,000
	2 Years Pre-diagnosis	$8,400±$16,000
	1 Year Pre-diagnosis	$23,500±$26,700
	1 Year Post-diagnosis	$30,300±$31,800
	2 Years Post-diagnosis	$20,500±$29,300
	3 Years Post-diagnosis	$17,100±$22,200
	4 Years Post-diagnosis	$18,100±$25,200
	5 Years Post-diagnosis	$17,800±$30,900
Keshishian et al. 2018 (USA) [4]	IBM	$52,800
Capkun et al. 2017 (USA)	IBM	$38,300±$4,100

Costs have been converted to 2023 USD with adjustments for purchasing power parity.

* Costs include lost productivity

DM: Dermatomyositis, IBM: Inclusion Body Myositis, IIM: Idiopathic Inflammatory Myositis, PM: Polymyositis

### Total aggregated costs of IIM

**Fig 2:**
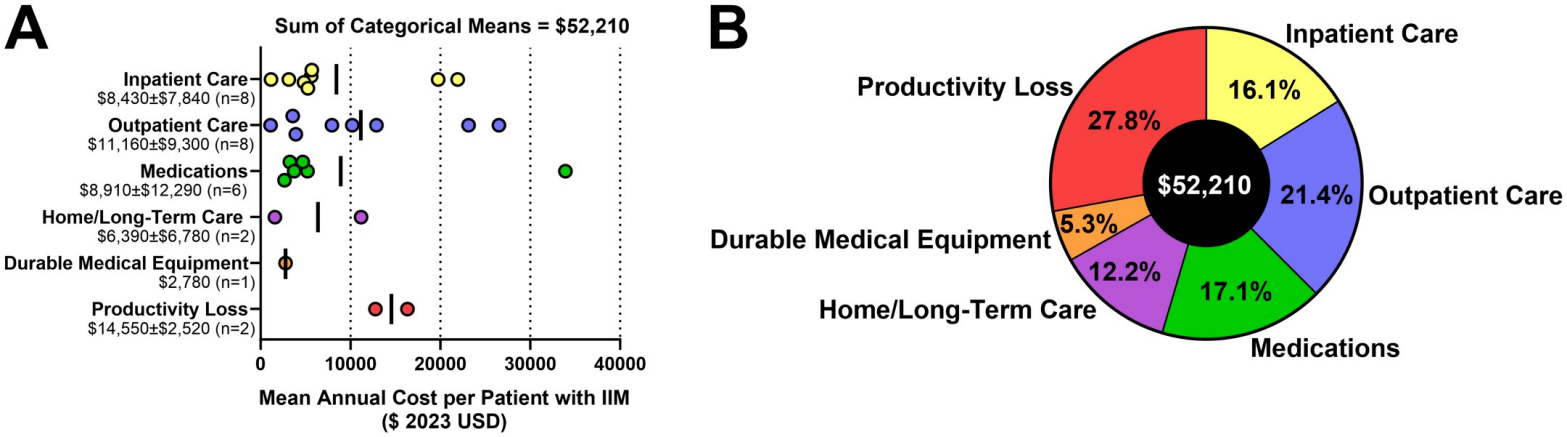
Aggregate cost estimate of IIM per patient per year. (A) Categorized mean annual costs of IIM obtained from all studies included in this review (expressed in 2023 USD with adjustments for purchasing power parity applied). Vertical bars indicate the means of each cost category. (B) Adding the means of each cost category provides an estimate for the total cost of IIM per patient per year of $52,210, with an important caveat that costs varied substantially both within and between studies identified in this review. IIM: Idiopathic Inflammatory Myopathies USD: United States of America dollars.

Major direct cost categories identified included inpatient care, outpatient care (including emergency and urgent care visits), medication, home/long-term care, and durable medical equipment. Major indirect cost categories identified included lost productivity (long- and short-term), and impaired non-work activity. The studies meeting eligibility criteria for this systematic review used heterogeneous research methods, patient populations, and study designs, with no individual study capturing all major cost components. A key aim of this review was to synthesize an estimate of total cost of IIM per patient per year. Given the heterogeneity in data reporting, a cost estimate was generated by taking the cost-per-annum data extracted from individual studies were charted according to their best-aligned major cost category (Fig 2A), and the means of each cost category were added together. By this calculation, the total societal cost of IIM was estimated to be $52,210 per annum per patient with IIM (Fig 2B). Reductions in quality of life were not monetized and are not reflected in the aggregate total. Though not specifically quantified in this review, it should be appreciated that considerable cost heterogeneity exists between individuals which will be affected by characteristics such as age, sex, employment status, disease subtype, time since diagnosis, prescribed treatment, place of residence, ability level, and the amount/type of formal and informal care required.

## Discussion

We found that the aggregated cost of IIM was $52,210 per patient per year (2023 USD). Total costs of IIM encompassed productivity losses among working-age individuals (27.8% of total costs), outpatient care (21.4% of total costs), medications (17.1% of total costs), inpatient care (16.1% of total costs), home/long-term care (12.2% of total costs), and durable medical equipment (5.3% of total costs).

IVIg was both a major cost and a major source of cost variability. One study reported use of IVIg as first-line therapy for IIM, and had annualized per patient medication costs of $33,909 [42], whereas mean medication costs were $3,908 ± $1,042 in other studies [4, 9, 26, 27, 29]. With a rapidly changing diagnostic and treatment landscape (*e*.*g*., increasing use of IVIg/SCIg and emergence of chimeric antigen receptor T-cell therapies [17]), updated studies are required to reflect the current costs of IIM. Only three studies [9, 28, 42] identified in this review provided data coverage more recent than 2014, and no study provided data coverage newer than 2019. IVIg treatments, frequently received bi-weekly or monthly [12, 13], cost approximately $10,000 per infusion in the USA, and depending on the geographical hospital location, could cost 1.5–2.0 times more [13]. At up to $30,000 per patient per month, the average cost of IVIg per patient with IIM could reach $250,000 [14]. IVIg is now used, not only as a rescue therapy, but more commonly as a first-line therapy in treating patients with IIMs [12, 48]. For example, Miyazaki *et al*. [42] described immunoglobulins as a first-line therapy for IIM, and showed that drug costs were nearly 10-fold higher in the first year post-diagnosis than in the second year post-diagnosis [42]. In contrast, on average, the first use of Ig for IIM occurred 5.5 years after first diagnosis in Sweden [49], and the relatively low costs attributed to biologic immunosuppressants were observed to increase with each passing year after diagnosis [9]. Increased utilization of Ig would drastically increase the medication cost per patient, as well as outpatient costs, due to the healthcare infrastructure required to administer IVIg [50]. The advent of home-based SCIg has made IIM treatment more convenient for patients and can reduce outpatient costs compared to IVIg administered in clinic [51]. However, any savings must be weighed against a 30–50% increase in dosage due to the lower bioavailability of Ig delivered subcutaneously vs intravenously [52]. The high costs of some therapies may be insurmountable barriers in low-income nations not represented in this review. Studies comparing treatment response by IIM subtype are needed to guide efficient use of expensive treatments for IIM around the world.

Inpatient and outpatient costs were substantially higher in the USA than in other countries. Higher per patient spending in the USA has been reported for Parkinson’s disease [53] and on health care in general in comparison to other member countries of the Organisation for Economic Co-operation and Development (OECD) without a concomitant increase in healthcare utilization [54]. Notably, one study from the USA found that costs billed by the hospital were threefold higher than the costs incurred by the hospital for the same stay in IIM patients [37]; such cost inflation is not unique to IIM [55]. In contrast, a hospital cost-to-charge ratio of 0.73 was reported for Thailand [40]. Thus, cost inflation by hospitals should be considered a source of cost heterogeneity.

This review was limited in its ability to compare between IIM subtypes. With one exception [29], the studies were either focused on a single subtype, or pooled subtypes together. The higher reported cost of DM compared to PM and interstitial myositis [29] may be attributed to the higher association of DM with malignancy and multisystem involvement, requiring increased investigations, oncological treatment, and inpatient stays [10]. Cross-study comparisons were complicated by inconsistent categorization of costs, and differences in the types of costs reported. This variability is not entirely unexpected because the division of costs of care amongst public institutions, private entities, and individuals varies regionally [54]. Our choice to be inclusive regarding the type and source of IIM-associated cost data combined with our approach to data reduction precluded any stratification of costs according to patient demographic characteristics beyond those presented in the original studies. Many of the studies available reflect the outdated clinical classification system of IIMs. Recently, there has been an evolution of the diagnostic algorithm in IIMs, with many more IIM subtypes identified that impact the therapeutic regimen [56, 57]. For example, “polymyositis” has been largely replaced by other IIM entities, such as necrotizing myopathy and anti-synthetase syndrome [56]. An update to disease-specific billing codes for the evolving IIM subtypes would reduce barriers to understanding costs of IIM care using administrative data with uniform collection procedures.

Assessments of direct healthcare costs and lost productivity do not capture all aspects of disease burden on the individual, healthcare systems, or society [58]. Costs associated with missed or late diagnoses are not well represented. For example, medical costs increased substantially in the year prior to IIM diagnosis [9]. A lack of research examining patient perspectives on the impact of diagnostic delay has been noted previously [59]. Attempts to capture costs of IIM should include a pre-diagnosis period to help capture costs associated with delayed and misdiagnoses. Although productivity losses identified accounted for 27.8% of total costs in this review ($14,554), costs of IIM associated with reduced quality of life [8, 28], informal caregiving [60], and diminished productivity of individuals outside the labor force (e.g., retirees) were not monetized in the identified studies. Impairment in non-work activity was 40% for individuals with DM/PM [28]. Impairments in work and non-work activity can have a profound impact on the financial wellbeing and quality of life of patients and their families. One study, published after the search cutoff date, reported annualized costs of illness for IBM in Germany to be €75,985±€67,391 (€ 2021) per patient, with 41% attributed to the costs of informal caregiving, usually by a spouse [60]. The costs and burden of IIM will vary widely depending on disease severity, economic disparities, the availability of care and treatment options, and personal circumstances. Gaining complete understanding of IIM impacts from the perspective of patients and their families/care partner(s) is vitally important for cost effectiveness studies to weigh against the high costs of evolving therapies and thresholds for a society’s willingness to pay [61–64].

## Conclusion

The costs of IIM are substantial, estimated at $52,210 per patient year on average. Direct costs of formal inpatient, outpatient, nursing, and home care, durable medical equipment, and medications accounted for 72% of the total ($37,656). Indirect costs of IIM, which were limited to the lost productivity of working-age individuals with IIM, encompassed 28% of total costs ($14,554). However, available cost data largely reflect dated diagnostic criteria and treatment strategies. This review emphasizes the urgent need for updated cost of illness studies reflecting new clinical IIM subtypes and advancements in treatment strategies which can inform cost-effectiveness studies, which in turn, must consider the impact of IIM on patients as well as their families.

## Supporting information

S1 Table PRISMA 2020 guidelines checklist. (DOCX)

S2 Table MEDLINE (Ovid) search strategy. (DOCX)

S3 Table Embase (Ovid) search strategy. (DOCX)

S4 Table CINAHL (EBSCOhost) search strategy. (DOCX)

S5 Table Scopus search strategy. (DOCX)

S6 Table Summary of JBI critical appraisal checklists for economic evaluations. (DOCX)

S7 Table Summary of CHEERS reporting quality checklists. (DOCX)

S1 Fig Timeline of data coverage. (DOCX)

S1 File Data extraction form used to gather economic data. (DOCX)
