# Supplementary material for: Direct and indirect costs of idiopathic inflammatory myopathies in adults: A systematic review
Source: PLoS One. 2024 Jul 26;19(7):e0307144. doi: 10.1371/journal.pone.0307144 (PMC11280229; doi:10.1371/journal.pone.0307144)
Supplement: S2 Table — (DOCX) [file pone.0307144.s002.docx]

**S2 Table. MEDLINE (Ovid) search strategy**

| Ovid MEDLINE(R) ALL <1946 to April 19, 2023> | | |
| --- | --- | --- |
| **#** | **Searches** | **Results** |
| 1 | exp myositis/ | 22365 |
| 2 | (myositi* or myopath* or polymyositi* or dermatomyositi* or pyomyositi* or neuromyositi* or dermatomucomyositi* or poikilodermatomyositi* or fibromyositi* or inomyositi*).ti,ab,kf. | 51442 |
| 3 | (inflam* adj2 musc* disease*).ti,ab,kf. | 366 |
| 4 | ((IIM or IIMs) and (myo* or muscle* or muscul*)).ti,ab,kf. | 1117 |
| 5 | ((antisynthetase* or anti-synthetase*) adj2 syndrome*).ti,ab,kf. | 866 |
| 6 | ((wegner hepp unverrricht or muenchmeyer* or munchmeyer* or "man of stone") adj2 (disease* or syndrome*)).ti,ab,kf. | 29 |
| 7 | ((ossifica* or ossify*) adj3 (myasiti* or myo* or muscle* or muscul* or fibrodysplasia* or fibro-dysplasia* or neuro*)).ti,ab,kf. | 2870 |
| 8 | ((neuro* or charcot*) adj3 (osteoarthr* or paraosteoarthr*)).ti,ab,kf. | 600 |
| 9 | (neuroosteoarthr* or neurosteoarthr*).ti,ab,kf. | 21 |
| 10 | or/1-9 | 57243 |
| 11 | economics/ | 27498 |
| 12 | exp "costs and cost analysis"/ | 263838 |
| 13 | economics, nursing/ | 4013 |
| 14 | economics, medical/ | 9243 |
| 15 | economics, pharmaceutical/ | 3098 |
| 16 | exp economics, hospital/ | 25697 |
| 17 | economics, dental/ | 1920 |
| 18 | exp "fees and charges"/ | 31339 |
| 19 | exp budgets/ | 14098 |
| 20 | budget*.ti,ab,kf. | 35566 |
| 21 | (economic* or cost or costs or costly or costing or price or prices or pricing or pharmacoeconomic* or pharmaco-economic* or expenditure or expenditures or expense or expenses or financial or finance or finances or financed).ti,kf. | 277285 |
| 22 | (economic* or cost or costs or costly or costing or price or prices or pricing or pharmacoeconomic* or pharmaco-economic* or expenditure or expenditures or expense or expenses or financial or finance or finances or financed).ab. /freq=2 | 374015 |
| 23 | (cost* adj2 (effective* or utilit* or benefit* or minimi* or analy* or outcome or outcomes)).ab,kf. | 205657 |
| 24 | (value adj2 (money or monetary)).ti,ab,kf. | 2995 |
| 25 | exp models, economic/ | 16198 |
| 26 | economic model*.ab,kf. | 4142 |
| 27 | markov chains/ | 15931 |
| 28 | markov.ti,ab,kf. | 28589 |
| 29 | monte carlo method/ | 32067 |
| 30 | monte carlo.ti,ab,kf. | 59371 |
| 31 | exp decision theory/ | 13194 |
| 32 | (decision* adj2 (tree* or analy* or model*)).ti,ab,kf. | 36619 |
| 33 | or/11-32 | 884917 |
| 34 | 10 and 33 | 325 |
