# Supplementary material for: Direct and indirect costs of idiopathic inflammatory myopathies in adults: A systematic review
Source: PLoS One. 2024 Jul 26;19(7):e0307144. doi: 10.1371/journal.pone.0307144 (PMC11280229; doi:10.1371/journal.pone.0307144)
Supplement: S3 Table — (DOCX) [file pone.0307144.s003.docx]

**S3 Table. Embase (Ovid) search strategy**

| Embase Classic+Embase <1947 to 2023 April 19> | | |
| --- | --- | --- |
| **#** | **Searches** | **Results** |
| 1 | exp myositis/ | 51940 |
| 2 | (myositi* or myopath* or polymyositi* or dermatomyositi* or pyomyositi* or neuromyositi* or dermatomucomyositi* or poikilodermatomyositi* or fibromyositi* or inomyositi*).ti,ab,kf. | 80239 |
| 3 | (inflam* adj2 musc* disease*).ti,ab,kf. | 580 |
| 4 | ((IIM or IIMs) and (myo* or muscle* or muscul*)).ti,ab,kf. | 2325 |
| 5 | ((antisynthetase* or anti-synthetase*) adj2 syndrome*).ti,ab,kf. | 1659 |
| 6 | ((wegner hepp unverrricht or muenchmeyer* or munchmeyer* or "man of stone") adj2 (disease* or syndrome*)).ti,ab,kf. | 43 |
| 7 | ((ossifica* or ossify*) adj3 (myasiti* or myo* or muscle* or muscul* or fibrodysplasia* or fibro-dysplasia* or neuro*)).ti,ab,kf. | 3742 |
| 8 | ((neuro* or charcot*) adj3 (osteoarthr* or paraosteoarthr*)).ti,ab,kf. | 881 |
| 9 | (neuroosteoarthr* or neurosteoarthr*).ti,ab,kf. | 49 |
| 10 | or/1-9 | 97200 |
| 11 | economics/ | 247199 |
| 12 | cost/ | 65766 |
| 13 | exp health economics/ | 1037748 |
| 14 | budget/ | 34046 |
| 15 | budget*.ti,ab,kf. | 48321 |
| 16 | (economic* or cost or costs or costly or costing or price or prices or pricing or pharmacoeconomic* or pharmaco-economic* or expenditure or expenditures or expense or expenses or financial or finance or finances or financed).ti,kf. | 353637 |
| 17 | (economic* or cost or costs or costly or costing or price or prices or pricing or pharmacoeconomic* or pharmaco-economic* or expenditure or expenditures or expense or expenses or financial or finance or finances or financed).ab. /freq=2 | 533006 |
| 18 | (cost* adj2 (effective* or utilit* or benefit* or minimi* or analy* or outcome or outcomes)).ab,kf. | 290677 |
| 19 | (value adj2 (money or monetary)).ti,ab,kf. | 4124 |
| 20 | statistical model/ | 173192 |
| 21 | economic model*.ab,kf. | 6233 |
| 22 | probability/ | 150608 |
| 23 | markov.ti,ab,kf. | 38271 |
| 24 | monte carlo method/ | 50405 |
| 25 | monte carlo.ti,ab,kf. | 63407 |
| 26 | decision theory/ | 1882 |
| 27 | decision tree/ | 21118 |
| 28 | (decision* adj2 (tree* or analy* or model*)).ti,ab,kf. | 51172 |
| 29 | or/11-28 | 2027207 |
| 30 | 10 and 29 | 1679 |
