# Supplementary material for: Direct and indirect costs of idiopathic inflammatory myopathies in adults: A systematic review
Source: PLoS One. 2024 Jul 26;19(7):e0307144. doi: 10.1371/journal.pone.0307144 (PMC11280229; doi:10.1371/journal.pone.0307144)
Supplement: S4 Table — (DOCX) [file pone.0307144.s004.docx]

**S4 Table. CINAHL (EBSCOhost) search strategy**

| **#** | **Searches** | **Results** |
| --- | --- | --- |
| S1 | (MH "Myositis+") | 4,332 |
| S2 | TI ( myositi* or myopath* or polymyositi* or dermatomyositi* or pyomyositi* or neuromyositi* or dermatomucomyositi* or poikilodermatomyositi* or fibromyositi* or inomyositi* ) OR AB ( myositi* or myopath* or polymyositi* or dermatomyositi* or pyomyositi* or neuromyositi* or dermatomucomyositi* or poikilodermatomyositi* or fibromyositi* or inomyositi* ) | 8,539 |
| S3 | TI ( inflam* N2 "musc* disease*" ) OR AB ( inflam* N2 "musc* disease*" ) | 98 |
| S4 | TI ( (IIM or IIMs) and (myo* or muscle* or muscul*) ) OR AB ( (IIM or IIMs) and (myo* or muscle* or muscul*) ) | 322 |
| S5 | TI ( (antisynthetase* or anti-synthetase*) N2 syndrome* ) OR AB ( (antisynthetase* or anti-synthetase*) N2 syndrome* ) | 268 |
| S6 | TI ( ("wegner hepp unverrricht" or muenchmeyer* or munchmeyer* or "man of stone") N2 (disease* or syndrome*) ) OR AB ( ("wegner hepp unverrricht" or muenchmeyer* or munchmeyer* or "man of stone") N2 (disease* or syndrome*) ) | 2 |
| S7 | TI ( (ossifica* or ossify*) N3 (myasiti* or myo* or muscle* or muscul* or fibrodysplasia* or fibro-dysplasia* or neuro*) ) OR AB ( (ossifica* or ossify*) N3 (myasiti* or myo* or muscle* or muscul* or fibrodysplasia* or fibro-dysplasia* or neuro*) ) | 520 |
| S8 | TI ( (neuro* or charcot*) N3 (osteoarthr* or paraosteoarthr*) ) OR AB ( (neuro* or charcot*) N3 (osteoarthr* or paraosteoarthr*) ) | 247 |
| S9 | TI ( neuroosteoarthr* or neurosteoarthr* ) OR AB ( neuroosteoarthr* or neurosteoarthr* ) | 14 |
| S10 | S1 OR S2 OR S3 OR S4 OR S5 OR S6 OR S7 OR S8 OR S9 | 9,974 |
| S11 | MH "Economics" OR MH "Costs and Cost Analysis+" OR MH "Economic Aspects of Illness" OR MH "Resource Allocation+" OR MH "Economic Value of Life" OR MH "Economics, Pharmaceutical" OR MH "Economics, Dental" OR MH "Fees and Charges+" OR MH "Budgets" OR MH "Decision Trees" OR TI budget* OR TI ( economic* OR cost OR costs OR costly OR costing OR price OR prices OR pricing OR pharmacoeconomic* OR "pharmaco-economic*" OR expenditure OR expenditures OR expense OR expenses OR financial OR finance OR finances OR financed ) OR TI ( cost* N2 (effective* OR utilit* OR benefit* OR minimi* OR analy* OR outcome OR outcomes) ) OR TI ( value N2 (money OR monetary) ) OR TI ( markov OR monte carlo ) OR TI ( decision* N2 (tree* OR analy* OR model*) ) OR AB budget* OR AB ( economic* OR cost OR costs OR costly OR costing OR price OR prices OR pricing OR pharmacoeconomic* OR "pharmaco-economic*" OR expenditure OR expenditures OR expense OR expenses OR financial OR finance OR finances OR financed ) OR AB ( cost* N2 (effective* OR utilit* OR benefit* OR minimi* OR analy* OR outcome OR outcomes) ) OR AB ( value N2 (money OR monetary) ) OR AB ( markov OR monte carlo ) OR AB ( decision* N2 (tree* OR analy* OR model*) ) | 465,670 |
| S12 | S10 AND S11 | 156 |
