# Supplementary material for: Direct and indirect costs of idiopathic inflammatory myopathies in adults: A systematic review
Source: PLoS One. 2024 Jul 26;19(7):e0307144. doi: 10.1371/journal.pone.0307144 (PMC11280229; doi:10.1371/journal.pone.0307144)
Supplement: S5 Table — (DOCX) [file pone.0307144.s005.docx]

**S5 Table. Scopus search strategy**

| **#** | **Searches** | **Results** |
| --- | --- | --- |
| 1 | ( ( TITLE-ABS-KEY ( myositi* OR myopath* OR polymyositi* OR dermatomyositi* OR pyomyositi* OR neuromyositi* OR dermatomucomyositi* OR poikilodermatomyositi* OR fibromyositi* OR inomyositi* ) ) OR ( TITLE-ABS-KEY ( inflam* W/2 "musc* disease*" ) ) OR ( TITLE-ABS-KEY ( ( iim OR iims ) AND ( myo* OR muscle* OR muscul* ) ) ) OR ( TITLE-ABS-KEY ( ( antisynthetase* OR anti-synthetase* ) W/2 syndrome* ) ) OR ( TITLE-ABS-KEY ( ( "wegner hepp unverrricht" OR muenchmeyer* OR munchmeyer* OR "man of stone" ) W/2 ( disease* OR syndrome* ) ) ) OR ( TITLE-ABS-KEY ( ( ossifica* OR ossify* ) W/3 ( myasiti* OR myo* OR muscle* OR muscul* OR fibrodysplasia* OR fibro-dysplasia* OR neuro* ) ) ) OR ( TITLE-ABS-KEY ( ( neuro* OR charcot* ) W/3 ( osteoarthr* OR paraosteoarthr* ) ) ) OR ( TITLE-ABS-KEY ( neuroosteoarthr* OR neurosteoarthr* ) ) ) AND ( ( TITLE-ABS-KEY ( budget* OR economic* OR cost OR costs OR costly OR costing OR price OR prices OR pricing OR pharmacoeconomic* OR pharmaco-economic* OR expenditure OR expenditures OR expense OR expenses OR financial OR finance OR finances OR financed OR markov OR "monte carlo" ) ) OR ( TITLE-ABS-KEY ( value W/2 ( money OR monetary ) ) ) OR ( TITLE-ABS-KEY ( decision* W/2 ( tree* OR analy* OR model* ) ) ) ) | 2296 |
