# Supplementary material for: Direct and indirect costs of idiopathic inflammatory myopathies in adults: A systematic review
Source: PLoS One. 2024 Jul 26;19(7):e0307144. doi: 10.1371/journal.pone.0307144 (PMC11280229; doi:10.1371/journal.pone.0307144)
Supplement: S7 Table — (DOCX) [file pone.0307144.s007.docx]

**S8 Table: Summary of CHEERS reporting quality checklists**
