# Supplementary material for: Direct and indirect costs of idiopathic inflammatory myopathies in adults: A systematic review
Source: PLoS One. 2024 Jul 26;19(7):e0307144. doi: 10.1371/journal.pone.0307144 (PMC11280229; doi:10.1371/journal.pone.0307144)
Supplement: S1 Fig — (DOCX) [file pone.0307144.s008.docx]

**
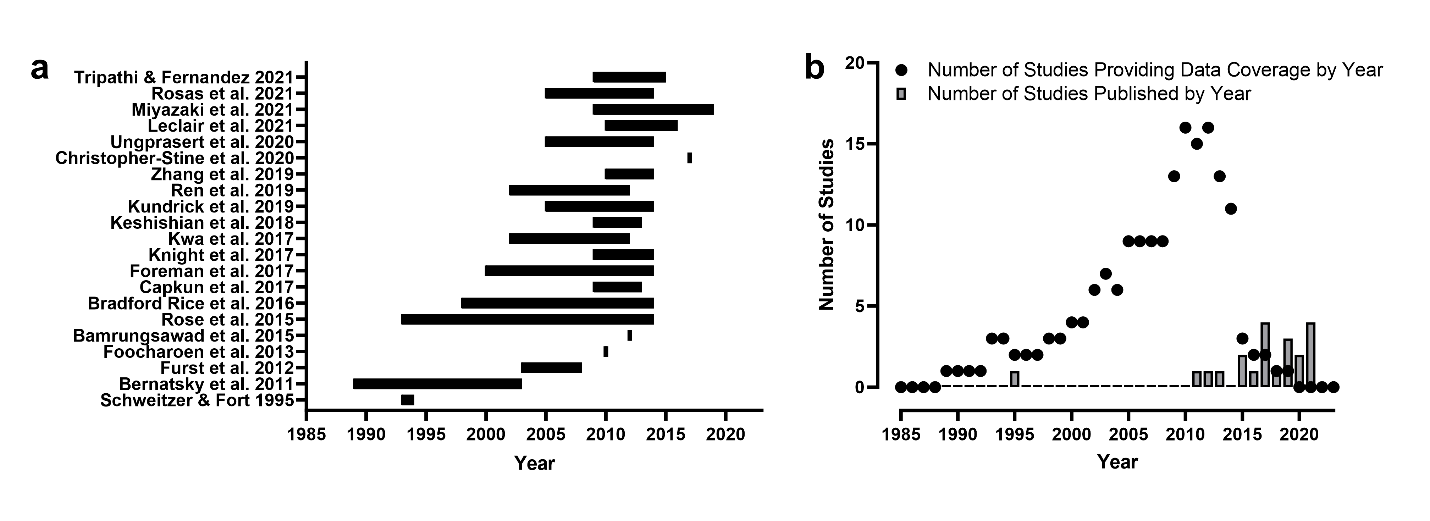
**

**S9 Figure.** **Timeline of data coverage.** a: Study-by-study depiction of years of data coverage. b: Summary of the number of studies with data coverage per year and the number of studies published per year.
