# Supplementary material for: Direct and indirect costs of idiopathic inflammatory myopathies in adults: A systematic review
Source: PLoS One. 2024 Jul 26;19(7):e0307144. doi: 10.1371/journal.pone.0307144 (PMC11280229; doi:10.1371/journal.pone.0307144)
Supplement: S1 File — (DOCX) [file pone.0307144.s009.docx]

**S6 Supplement. Data extraction form used to gather economic data**

Data Extraction Sheet: Cost of Inflammatory Myopathies

**Article Title:**

**Author:**

**Year Published:**

**Location:**

**Citation:**

**Baseline Characteristics**

**Study Overview:**

**Patient Characteristics:**

| Disease Focused On |  |
| --- | --- |
| Number of Participants |  |
| % Female |  |
| Average Age (SD) |  |

**Economic Study Design:**

**Perspective of Analysis:**

**Time Horizon of Analysis:**

**Comparator Group (If Applicable):**

| Number of Comparators |  |
| --- | --- |
| % Female |  |
| Average Age (SD) |  |

**Cost Analysis**

**Inpatient Costs**

| **Type** | **In Article? (Yes/No)** | **Cost**  **(currency, year)** | **Notes**  **(including data source)** |
| --- | --- | --- | --- |
| Overall Hospital Costs |  |  |  |
| Acute Care Hospitalizations |  |  |  |
| Length of Stay in Hospital |  |  |  |
| Patient Testing During Stay |  |  |  |
| Hospital Readmission Rates |  |  |  |

**Outpatient Costs**

| **Type** | **In Article? (Yes/No)** | **Cost**  **(currency, year)** | **Notes**  **(including data source)** |
| --- | --- | --- | --- |
| Overall Outpatient Cost |  |  |  |
| Physician Service: Hospital Outpatient Department Visits |  |  |  |
| Physician Service: Office Visits |  |  |  |
| Outpatient Procedure/Surgery |  |  |  |
| Diagnostic Services |  |  |  |
| Durable Medical Equipment (Assisted Device Usage) |  |  |  |

**Emergency Department/Urgent Care Costs**

| **Type** | **In Article? (Yes/No)** | **Cost**  **(currency, year)** | **Notes**  **(including data source)** |
| --- | --- | --- | --- |
| Overall ED/UC Cost |  |  |  |

**Medication Costs**

| **Type** | **In Article? (Yes/No)** | **Cost**  **(currency, year)** | **Notes**  **(including data source)** |
| --- | --- | --- | --- |
| Overall Medication Cost |  |  |  |
| IVIG |  |  |  |
| Corticosteroids: Prednisone or Methylprednisolone |  |  |  |
| Methotrexate |  |  |  |
| Mycophenolate Mofetil |  |  |  |
| Azathioprine |  |  |  |
| Rituximab |  |  |  |
| Repository Corticotropin Injection |  |  |  |

**Other Healthcare Costs**

| **Type** | **In Article? (Yes/No)** | **Cost**  **(currency, year)** | **Notes**  **(including data source)** |
| --- | --- | --- | --- |
| Overall “other” category |  |  |  |
| Skilled Nursing Facilities |  |  |  |
| Home Health Agency Visits |  |  |  |
| Hospice Care |  |  |  |
| Informal Care |  |  |  |
| Other: _______ |  |  |  |

**Direct Non-Medical Costs**

| **Type** | **In Article? (Yes/No)** | **Cost**  **(currency, year)** | **Notes**  **(including data source)** |
| --- | --- | --- | --- |
| Overall direct non-medical costs |  |  |  |
| Parking |  |  |  |
| Travelling to Appointments |  |  |  |
| Accommodations |  |  |  |
| Meals |  |  |  |
| Other: _______ |  |  |  |

**Indirect Costs**

| **Type** | **In Article? (Yes/No)** | **Cost**  **(currency, year)** | **Notes**  **(including data source)** |
| --- | --- | --- | --- |
| Overall Indirect Cost |  |  |  |
| Absenteeism |  |  |  |
| Presenteeism |  |  |  |
| Disability Pension |  |  |  |

**Overall Costs Assessed**

| **Type** | **In Article? (Yes/No)** | **Cost**  **(currency, year)** | **Notes**  **(including data source)** |
| --- | --- | --- | --- |
| Inpatient Costs |  |  |  |
| Outpatient Costs |  |  |  |
| ED/UC Costs |  |  |  |
| Medication Costs |  |  |  |
| Other Costs |  |  |  |
| Direct Non-Medical Costs |  |  |  |
| Indirect Costs |  |  |  |
| Total Costs |  |  |  |
